# Interlaboratory Comparison on Absolute Photoluminescence Quantum Yield Measurements of Solid Light Converting Phosphors with Three Commercial Integrating Sphere Setups

**DOI:** 10.1021/acs.analchem.4c00372

**Published:** 2024-04-17

**Authors:** Saskia Fiedler, Florian Frenzel, Christian Würth, Isabella Tavernaro, Michelle Grüne, Stefan Schweizer, Axel Engel, Ute Resch-Genger

**Affiliations:** † Division of Biophotonics, Federal Institute for Materials Research and Testing (BAM), Richard-Willstaetter-Strasse 11, D-12489 Berlin, Germany; § Faculty of Electrical Engineering, South Westphalia University of Applied Sciences, Lübecker Ring 2, 59494 Soest, Germany; ∥ Fraunhofer Application Center for Inorganic Phosphors, Branch Lab of Fraunhofer Institute for Microstructure of Materials and Systems IMWS, Lübecker Ring 2, 59494 Soest, Germany; ⊥ Schott AG Technical Services, Hattenbergstrasse 10, D-55122 Mainz, Germany

## Abstract

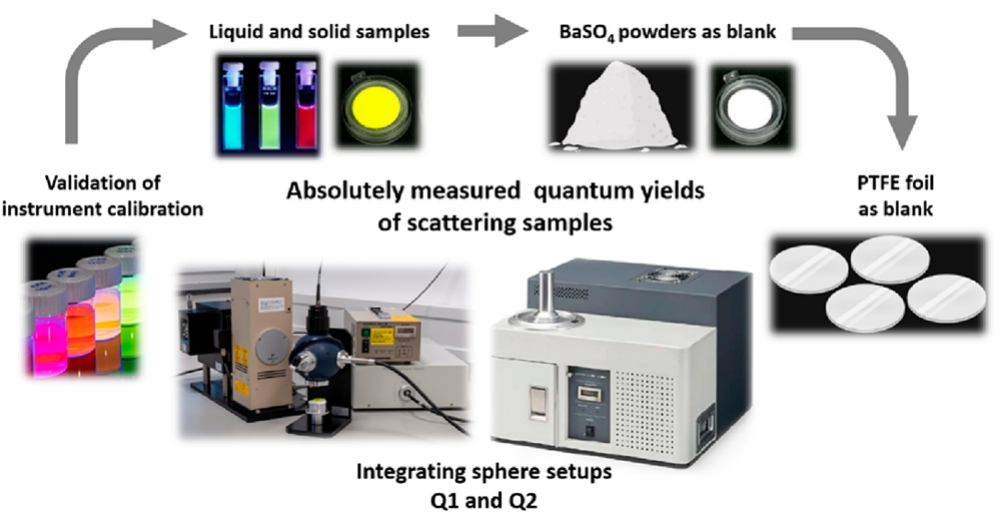

Scattering luminescent materials dispersed in liquid and solid matrices and luminescent powders are increasingly relevant for fundamental research and industry. Examples are luminescent nano- and microparticles and phosphors of different compositions in various matrices or incorporated into ceramics with applications in energy conversion, solid-state lighting, medical diagnostics, and security barcoding. The key parameter to characterize the performance of these materials is the photoluminescence/fluorescence quantum yield (Φ_f_), i.e., the number of emitted photons per number of absorbed photons. To identify and quantify the sources of uncertainty of absolute measurements of Φ_f_ of scattering samples, the first interlaboratory comparison (ILC) of three laboratories from academia and industry was performed by following identical measurement protocols. Thereby, two types of commercial stand-alone integrating sphere setups with different illumination and detection geometries were utilized for measuring the Φ_f_ of transparent and scattering dye solutions and solid phosphors, namely, YAG:Ce optoceramics of varying surface roughness, used as converter materials for blue light emitting diodes. Special emphasis was dedicated to the influence of the measurement geometry, the optical properties of the blank utilized to determine the number of photons of the incident excitation light absorbed by the sample, and the sample-specific surface roughness. While the Φ_f_ values of the liquid samples matched between instruments, Φ_f_ measurements of the optoceramics with different blanks revealed substantial differences. The ILC results underline the importance of the measurement geometry, sample position, and blank for reliable Φ_f_ data of scattering the YAG:Ce optoceramics, with the blank’s optical properties accounting for uncertainties exceeding 20%.

## Introduction

Luminescence methods such as fluorescence spectroscopy, microfluorometry, and fluorescence microscopy utilizing molecular and nanoscale luminescent reporters are broadly applied in the materials and life sciences.^1−5^ Applications range from sensing and bioimaging to barcoding, solid-state lighting, and energy conversion. Performance parameters of luminescent reporters include the spectral position of the luminophore absorption and emission bands, their spectral widths and overlap, as well as fundamental spectroscopic quantities acting as measures for the absorption and emission efficiency such as the molar absorption coefficient or absorption cross section and the photoluminescence or fluorescence quantum yield (Φ_f_).^6^ The latter equals the ratio of the number of emitted and absorbed photons, providing the conversion efficiency of absorbed into emitted photons.^7,8^ From the material or sample side, the size of the measurable fluorescence signal is determined by the product of the luminophore’s absorption coefficient or absorption cross section at the excitation wavelength and Φ_f_, termed brightness.^6^ Therefore, Φ_f_ is frequently used to select optimum emitters for applications, e.g., in medical diagnostics, solid-state lighting, and converter materials for light-emitting diodes (LEDs) and solar concentrators.^3,6,9−21^ Also, Φ_f_ measurements are an essential part of photophysical and mechanistic studies and provide the basis for the design of next-generation functional luminescent materials. This underlines the importance of reliable and reproducible Φ_f_ measurements for the scientific community, manufacturers, and users of commercial luminescent materials, as well as international standardization organizations like the International Electrotechnical Commission (IEC).

The Φ_f_ of transparent luminophore solutions can be determined relative to a so-called fluorescence quantum yield standard of known Φ_f_ using a conventional photometer and fluorescence spectrometer. However, the determination of Φ_f_ of scattering liquid and solid samples, such as dispersions of scattering luminescent particles, solid phosphors, and optoceramics, requires absolute measurements with an integrating sphere (IS).^22^ This triggered the renaissance of IS spectroscopy^23−29^ and the development of stand-alone IS setups and IS accessories for many spectrofluorometers in the past decade. In parallel, many recommended Φ_f_ standards^7,30,31^ were critically evaluated and protocols for relative and absolute Φ_f_ measurements of transparent luminescent materials were developed.^22,23,32−36^ Also, typical sources of error and achievable measurement uncertainties were addressed and quantified.

The need for reliable, comparable, and standardized Φ_f_ measurements expressed by companies involved in solid-state lighting and display technologies and/or the production and application of luminescent converter materials led to the first international standard IEC 62607-3-1 “Nanomanufacturing – Key control characteristics, Part 3-1: Luminescent nanomaterials – Quantum efficiency”, released in 2014. As a response to this need and to improve the reliability of Φ_f_ data, BAM certified a set of 12 dye-based Φ_f_ standards for the ultraviolet (UV), visible (vis), and near-infrared (NIR) region in 2022.^37^ These Φ_f_ standards, utilized as transparent dye solutions, are designed for relative Φ_f_ measurements of transparent luminescent samples and the performance validation of IS setups.^37^ However, at present, there are no scattering Φ_f_ standards that are available.

Aiming for the determination of sources of uncertainty of Φ_f_ measurements of scattering samples together with the need to update the standard IEC 62607-3-1, we performed a first interlaboratory comparison (ILC) of absolute Φ_f_ measurements with commercial stand-alone IS setups. This ILC involved three laboratories from academia and industry. This included BAM as a designated metrology institute and producer of reference materials such as fluorescence standards, Fraunhofer Application Center for Inorganic Phosphors at the Campus Soest of the South Westphalia University of Applied Sciences (FH SWF), with longstanding expertise in the development and spectroscopic characterization of functional luminescent materials, and SCHOTT AG (Schott). Schott produces optical materials for applications in automotive, lighting, health care, optical, and semiconductor technologies. To identify typical sources of uncertainty and quantify achievable measurement uncertainties, this ILC included the following steps: (i) assessment of the reliability of the spectral correction curves provided by the instrument manufacturer with validated and BAM-certified spectral fluorescence standards,^38,39^ (ii) absolute Φ_f_ measurements of transparent dye solutions and dye solutions containing defined amounts of scattering silica (SiO_2)_ particles, and (iii) absolute Φ_f_ measurements of industry-relevant scattering optoceramics of varying surface roughness. All measurements were performed according to the same measurement protocols using the same samples provided by BAM and Schott. Special emphasis was dedicated to the influence of the illumination and detection geometries of the IS setups, the sample-specific surface roughness, and the scattering and reflectance properties of the chosen nonluminescent blank on the resulting Φ_f_ values.

## Experimental Section

### Materials

#### Dyes

BAM-certified spectral fluorescence standards F003, F004, and F005^40^ were used to validate the instrument calibration, and BAM-certified Φ_f_ standards F015, F016, F017, and F019 were used to evaluate the Φ_f_ measurements.^37^

#### Solvent

Spectroscopic-grade ethanol, 99.9% purity, was obtained from LABSOLUTE.

#### Scatterer

Amorphous, nonporous SiO_2_ particles (300 nm) were added to transparent dye and blank solutions in defined amounts to introduce scattering. The synthesis and characterization of the nonfluorescent SiO_2_ particles are provided in the Supporting Information (SI).

#### Blanks

BaSO_4_ powders B1 from Sigma-Aldrich (99.99% trace metals basis), B2 (Puratronic, 99.998%, LOT 24177), and B3 (Puratronic, 99.998%, LOT 10226568) from Alfa Aesar (Thermo Fisher Scientific) and two PTFE diffuser targets from SphereOptics with thicknesses of 250 μm (SG 3203) and 2 mm (SG 3213) were used. The latter was polished on one side to allow for blank measurements with a smooth and a rough surface. The targets were cut into circles with a diameter of 15 mm to fit into the quartz Petri dish.

#### Optoceramic (OC) Sample

A YAG:Ce OC ((Y_1–*y*_Ce_*y*_)_3_Al_5_O_12_ (*y* = 0.001 to 0.01)) was provided by Schott. More details on sample preparation and measurement conditions are given in the SI.

## Results and Discussion

### Comparison of Instrumentation

**Figure 1:**
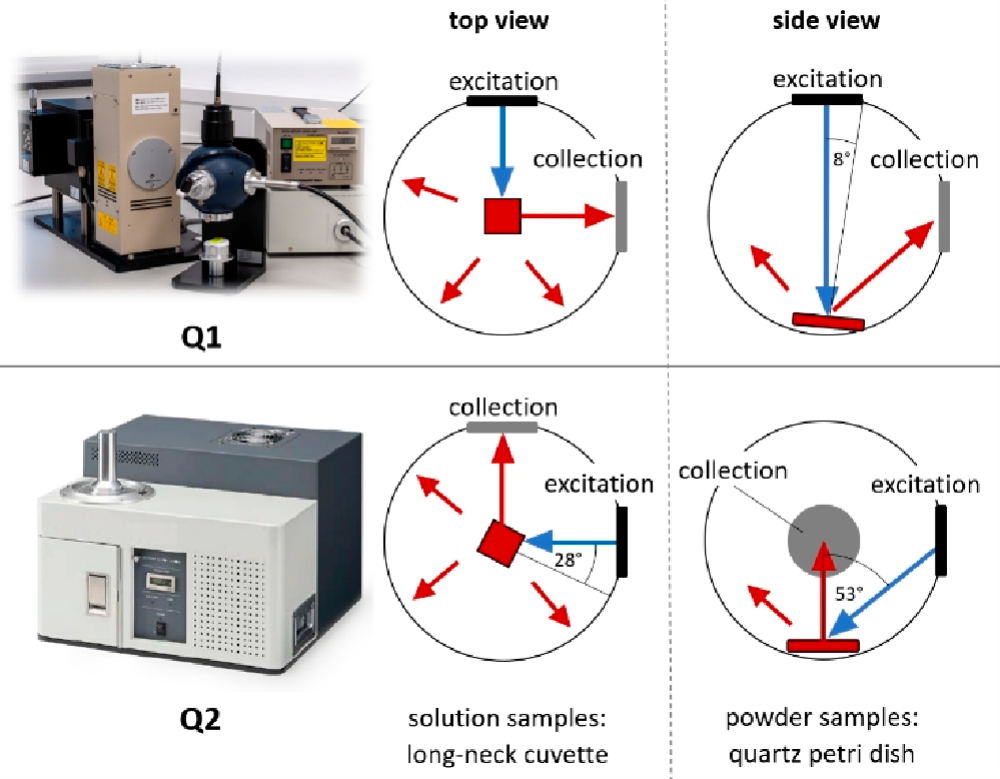
Overview of excitation and collection pathways of the IS setups Q1 (C9920-02G; Schott & FH SWF) and Q2 (C11347-11; BAM).

In this ILC, Quantaurus first (C9920-02G, “Q1”) and second generation (C11347-11, “Q2”) IS setups from Hamamatsu were used for absolute Φ_f_ measurements. The major differences between Q1 and Q2 are (i) the sample orientation within the IS and (ii) the illumination geometry, specifically, the angle of incidence of the excitation light on the sample surface and the corresponding reflections. The latter is dependent on the measured sample. For solutions, the sample is positioned in the center of the IS for both setups. In Q1, the sample is oriented perpendicular to the excitation beam, and it is positioned at a 28° angle to the excitation (Figure 1, central panels) in Q2.

Powders and solid samples are placed at the bottom of the IS with an 8° tilt to the excitation path in Q1 (Figure 1, top right panel). For Q2, such samples are positioned flat on the bottom of the IS resulting in a 53° angle to the excitation beam (Figure 1, bottom right panel). These differences and other less relevant ones are summarized in Table S1.

### Validation of the Setup Calibration Implemented by the Instrument Manufacturer

**Figure 2:**
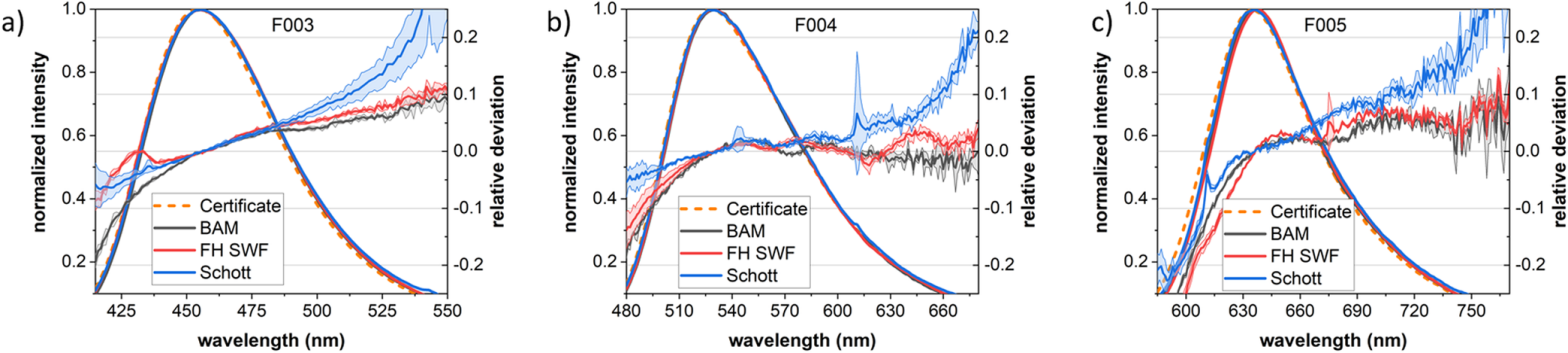
Validation of the spectral responsivity of Q1 and Q2 at FH SWF (red), Schott (blue), and BAM (black) by comparing the averaged (*N* = 4 × 2 × 3) F003 (a), F004 (b), and F005 (c) emission spectra measured for an absorbance of 0.1 (OD 0.1) at the recommended excitation wavelengths of 377, 423, and 553 nm with the corresponding certified emission spectra (orange).

The first step to accurate and comparable Φ_f_ measurements is reliable instrument calibration. Thus, the reliability of the wavelength (relative) dependent spectral responsivity (emission correction) of the different IS setups was assessed and validated in the ILC-relevant wavelength range of 400 to 750 nm using the BAM KIT dyes F003–F005, a commercial set of certified spectral fluorescence standards. Figure 2 displays the measured corrected emission spectra of F003–F005, including the relative standard deviations calculated from the averaged measurement repetitions at absorbances of 0.1 (indicated by shaded areas). The data of all ILC partners and the corresponding certified spectra of F003–F005 are in good agreement and also closely match the dyes’ emission spectra previously determined in an ILC on spectral correction.^38,39^ The small deviations from the certified values, obtained by dividing the measured spectra by the certified spectra of each dye, are more pronounced for shorter wavelengths. Within the spectral width of the emission band of each dye, the relative deviations are within ±0.1, confirming the reliability and comparability of the calibration of the IS setups.

### Comparability of the Φ_f_ Measurements of Transparent Dye Solutions

Φ_f_ measurements of four transparent dye solutions were performed using the certified Φ_f_ standards F015, F016, F017, and F019,^37^ varying in the spectral overlap between absorption and emission band and thus their sensitivity to reabsorption effects^41^ at two dye concentrations (OD 0.05 and OD 0.1 at λ_exc_). The Φ_f_ values and measurement uncertainties (standard deviation of *N* = 4 × 2 × 3) as well as the certified Φ_f_ values of each dye are shown in Table 1 and Table S2. The uncertainties given in these tables do not contain contributions from the calibration uncertainty^41^ of the IS setups. The obtained Φ_f_ data always match well with the certified Φ_f_ values, considering the respective measurement uncertainties. This comparison underlines the reliability of the calibration of all IS setups with larger uncertainties observed for setup Q1 compared with Q2.

**Table 1:** Averaged (*N* = 4 × 2 × 3) Φ_f_ Values (%) and Standard Deviations of Dispersions of the Certified Φ_f_ Standards F015 (λ_exc_ = 500 nm) and F016 (λ_exc_ = 400–420 nm) Containing Defined Amounts of 300 nm SiO_2_ Particles

dye + SiO_2_	BAM	FH SWF	Schott	certificate*
F015 + SiO_2_	92.5 ± 0.2	94.6 ± 2.2	91.3 ± 1.0	
F015	91.8 ± 1.6	94.5 ± 2.7	93.4 ± 0.8	96 ± 5.0
F016 + SiO_2_	56.0 ± 0.1	58.1 ± 0.9	55.9 ± 1.4	
F016	56.1 ± 0.2	58.4 ± 2.0	57.2 ± 1.7	59 ± 4.0

### Absolute Determination of Φ_f_ of Scattering Dye–SiO_2_ Particle Dispersions

To explore the effect of scatterers on the Φ_f_ determination, defined amounts of 300 nm SiO_2_ particles were added to ethanolic solutions of phosphates F015 and F016. As blanks, ethanol containing the same amount of SiO_2_ particles were applied, thus considering scattering-induced changes in the distribution of the incident and emitted photons. To rule out a possible quenching of dye fluorescence by SiO_2_ particles, time-resolved fluorescence measurements of the dye solutions without and with scatterers were performed prior to the Φ_f_ measurements by BAM. The matching fluorescence decay curves and lifetimes shown in Figure S2 confirm the absence of fluorescence quenching. All Φ_f_ obtained by the ILC partners are presented in Table 1.

The Φ_f_ values of the transparent and scattering dye solutions are in good agreement and match the certified Φ_f_ values. A prerequisite for this good comparability and the correct determination of the number of absorbed photons at the excitation wavelength is the consideration of scattering-induced changes in light distribution within the IS by matching the scattering properties of sample and blank, i.e., keeping the size and amount of the SiO_2_ particle scatterers for the sample and blank constant.

### YAG:Ce Optoceramics (OC)

Solid OC made from a scattering polycrystalline inorganic material (Y_1–*y*_Ce_*y*_)_3_Al_5_O_12_ (*y* = 0.001 to 0.01) with a very high Φ_f_, a high temperature stability, and an excellent long-term stability is a key functional material in modern lighting technologies. Applications are, e.g., converter materials for blue LEDs in the automotive industry. Φ_f_ is a key quantity for OC light conversion efficiency and performance. Despite the considerable application relevance, the comparability of Φ_f_ measurements of these materials across instruments and laboratories has not yet been assessed. In this first ILC we explored (i) the influence of the illumination and detection geometry of the IS setup, (ii) the choice of a suitable blank, and (iii) the influence of the sample-specific parameter “surface roughness” on the reliability and comparability of the resulting Φ_f_ values. Thus, we chose YAG:Ce OC samples with a Φ_f_ close to unity as this facilitates the detection of small Φ_f_ differences and allows for the straightforward identification of Φ_f_ values that are physically not meaningful, i.e., exceeding 100%.

In this ILC, we absolutely determined the Φ_f_ of YAG:Ce OC samples with varying surface roughness using previously developed and well-documented measurement protocols (SI). The samples were placed in a small quartz dish on the sample holder, which is part of the IS surface in both the Q1 and Q2 setups (Figure 1). Blank measurements are commonly done with an empty IS as the sample holder coated with the same material, i.e., Spectralon, as the IS surface, making it an ideal Lambertian diffusor with reflectivity of 99%.^42^ However, the sample holder surface can be prone to aging and contamination by absorbing and/or luminescent impurities, e.g., from previous samples. This can change its scattering characteristics and result in a nonideal scattering behavior. The presence of absorbing impurities can lead to an underestimation of the light fraction absorbed by the sample as the area of the excitation part of the sample’s spectrum is subtracted from that of the blank; as a result, Φ_f_ is overestimated. Thus, using an additional blank with scattering and transmission properties closely matching those of the sample is recommended and a prerequisite for an identical or at least similar distribution of the excitation light for sample and blank.

**Figure 3:**
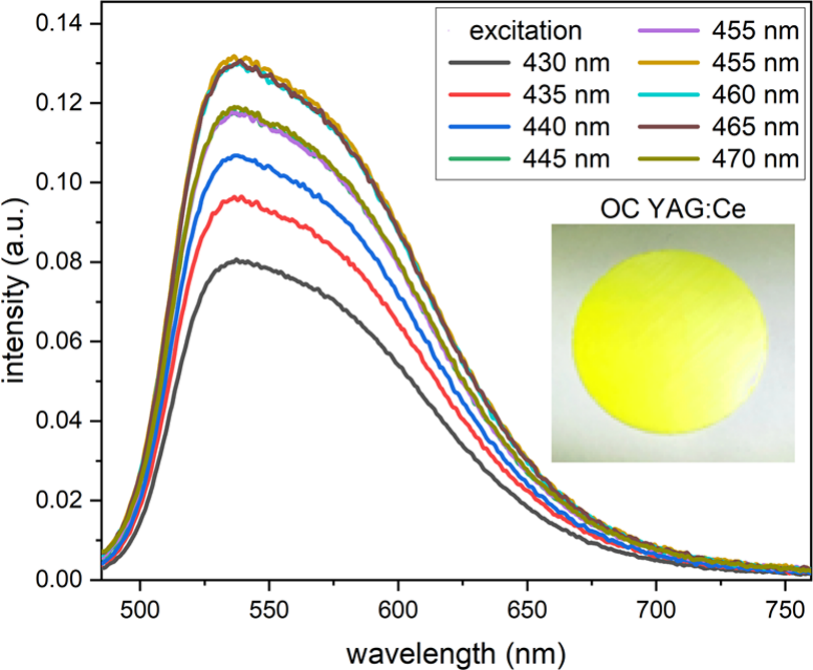
Emission spectra of the YAG:Ce OC as a function of excitation wavelength (λ_exc_ = 430–470 nm). Inset: photo of the polished OC sample.

Also, the light distribution in the IS can be affected by the sample’s/blank’s surface roughness, as the first reflex of the excitation light can considerably influence the measured fraction of absorbed photons and hence Φ_f_. To underline the importance of the blank’s optical properties, we performed Φ_f_ measurements of the same YAG:Ce OC with different types of blanks in Q1 and Q2. First, to identify the optimum excitation wavelength, the absorption and emission intensities of the YAG:Ce OC were measured as a function of excitation wavelength from 430 to 470 nm in 5 nm increments using a thin PTFE foil blank. As shown in Figure 3, the highest emission intensities resulted for excitation wavelengths between 445 and 465 nm. However, the emission spectra and Φ_f_ values are independent of excitation wavelength within the derived measurement uncertainties (see Figure 3 and Figure S4). All Φ_f_ measurements were performed at 455 nm, matching the output wavelength of blue LEDs.

### Influence of Blank for Q2

Prior to the ILC, different blanks were tested at BAM: BaSO_4_ powders B1, B2, and B3, and a thin PTFE foil, chosen due to their high and nearly wavelength-independent reflectivity in the visible region. Φ_f_ measurements were performed with and without the lid of the Petri dish to realize (i) a rough surface without a specular reflex (powders of different grain size without a lid), (ii) a surface with specular reflectivity (with a lid), and (iii) a flat surface with Lambertian scattering (PTFE foil without a lid). The YAG:Ce OC was always placed in a Petri dish without a lid.

**Figure 4:**
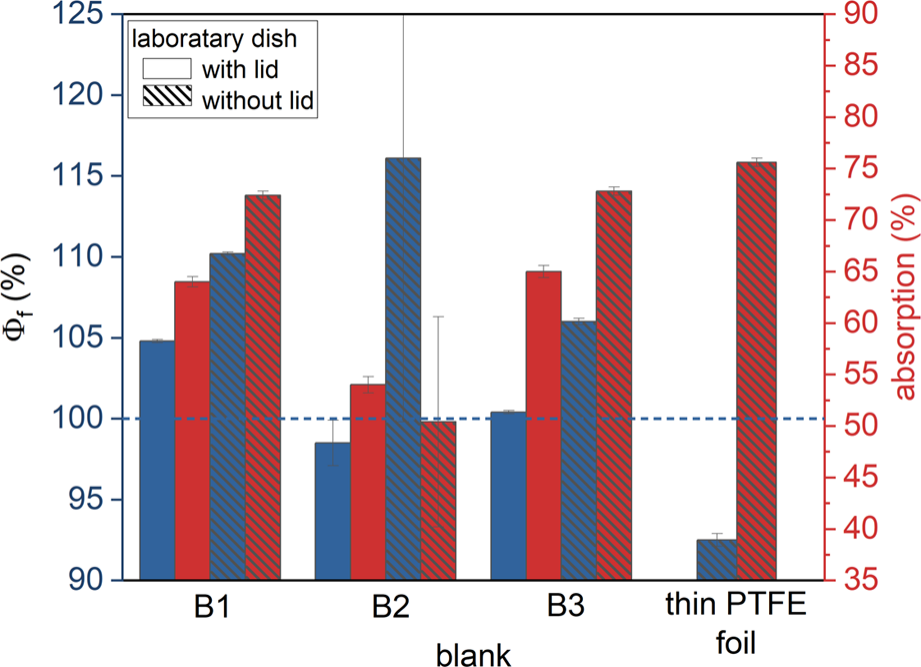
Absorption (red) and Φ_f_ (blue) of the YAG:Ce OC measured in a quartz Petri dish without a lid with Q2. Blanks: BaSO_4_ powders B1, B2, and B3 and a 250 μm thick PTFE foil, both with (solid) and without (shaded) a lid. λ_exc_ = 455 nm. All blanks were placed in the quartz Petri dish with a lid (solid bars) and without a lid (shaded bars). The YAG:Ce OC was always placed in the Petri dish without a lid.

For measurements using B1, B2, and B3 with and without a lid, physically meaningless and hence erroneous Φ_f_ values >100% were obtained, as shown in Figure 4. This indicates considerable differences in the scattering and reflection behaviors of the OC sample and the BaSO_4_ powder blanks. Likely explanations are differences in surface roughness and scattering behavior, as well as absorption characteristics caused by water adhesion during the fabrication process of the powders. This also explains the large Φ_f_ deviations of the supposedly identical powders B2 and B3 (the same manufacturer, different batches). As suggested by the more reasonable Φ_f_ values of (98.5 ± 1.4)% and (92.5 ± 0.4)% obtained for B2 and the thin PTFE foil, both materials present suitable blanks. Subsequently in the ILC, these two blanks were used to explore the influence of different instruments, measurement geometries, and sample handling procedures, as well as the influence of the OC surface roughness.

### ILC Results

**Figure 5:**
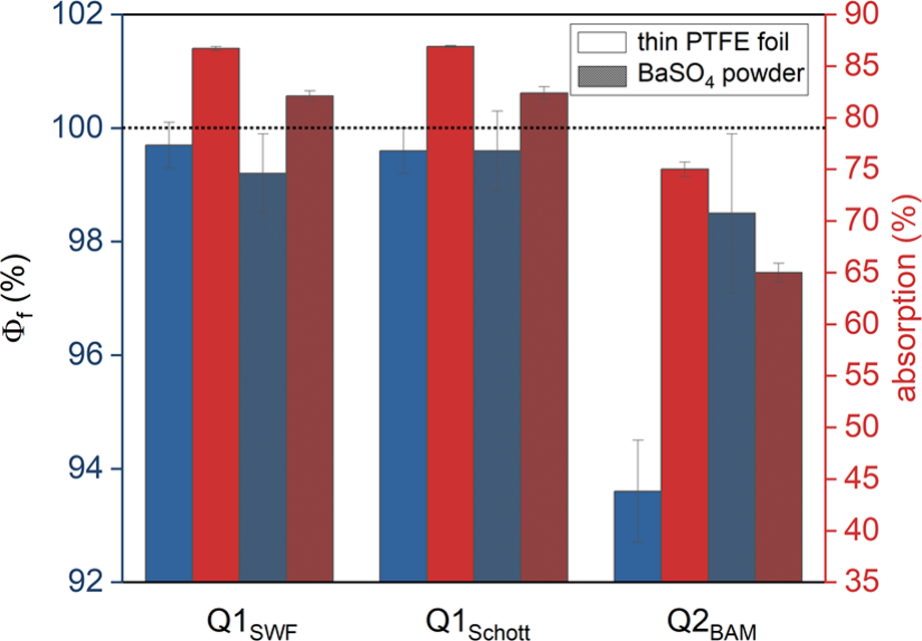
Averaged (*N* = 4 × 2 × 4) absorption (red) and Φ_f_ (blue) of the YAG:Ce OC, obtained with Q1 (FH SWF, Schott) and Q2 (BAM) using a thin PTFE foil (solid) and B2 BaSO_4_ powder with a lid (dotted) as a blank, respectively.

The results of the Φ_f_ measurements of the YAG:Ce OC with Q1 and Q2 using B2 and the thin PTFE foil as blanks are shown in Figure 5. Both Φ_f_ data sets collected with setup Q1 closely match, with no observable blank influence. For B2, we obtained Φ_f_ values of (99.2 ± 0.7)% (FH SWF) and (99.6 ± 0.7)% (Schott), and for the thin PTFE foil, (99.7 ± 0.4)% (FH SWF) and (99.6 ± 0.4)% (Schott). The statistical data analysis also included individual measurements with Φ_f_ values exceeding 100%. Measurements with Q2 yielded significantly lower Φ_f_ values and deviations in Φ_f_ for both blanks, i.e., Φ_f_ values of (93.6 ± 0.9)% and (98.5 ± 1.2)% for B2 and the PTFE foil, respectively. The observed deviations in Φ_f_ values for Q1 and Q2 cannot be attributed to the instrument calibration, as the corrected emission spectra and the Φ_f_ values of the transparent dye solutions measured with the three setups were in good agreement, also with the certificate (cf. the Validation and Comparability sections).

A more likely explanation for the considerable deviations in Φ_f_ is a different light distribution within the IS for the scattering samples resulting from the different measurement and detection geometries of setups Q1 and Q2. As shown in Figure 1, for Q1, an almost perpendicular sample excitation is realized (8° to the surface normal), while in the case of Q2, the excitation light hits the sample at an angle of 53° with respect to the sample surface normal. This small geometric difference between the setups affects the sensitivity to the blank surface structure. Evidently, the use of powder blanks is more error prone and can introduce higher, additional (handling) uncertainties, as control of the powder distribution within the Petri dish even with a lid to smoothen the surface is challenging. We thus recommend the usage of a solid blank like the PTFE foil for Φ_f_ measurements of solid samples.

### Influence of Sample and Blank Surface Properties

**Figure 6:**
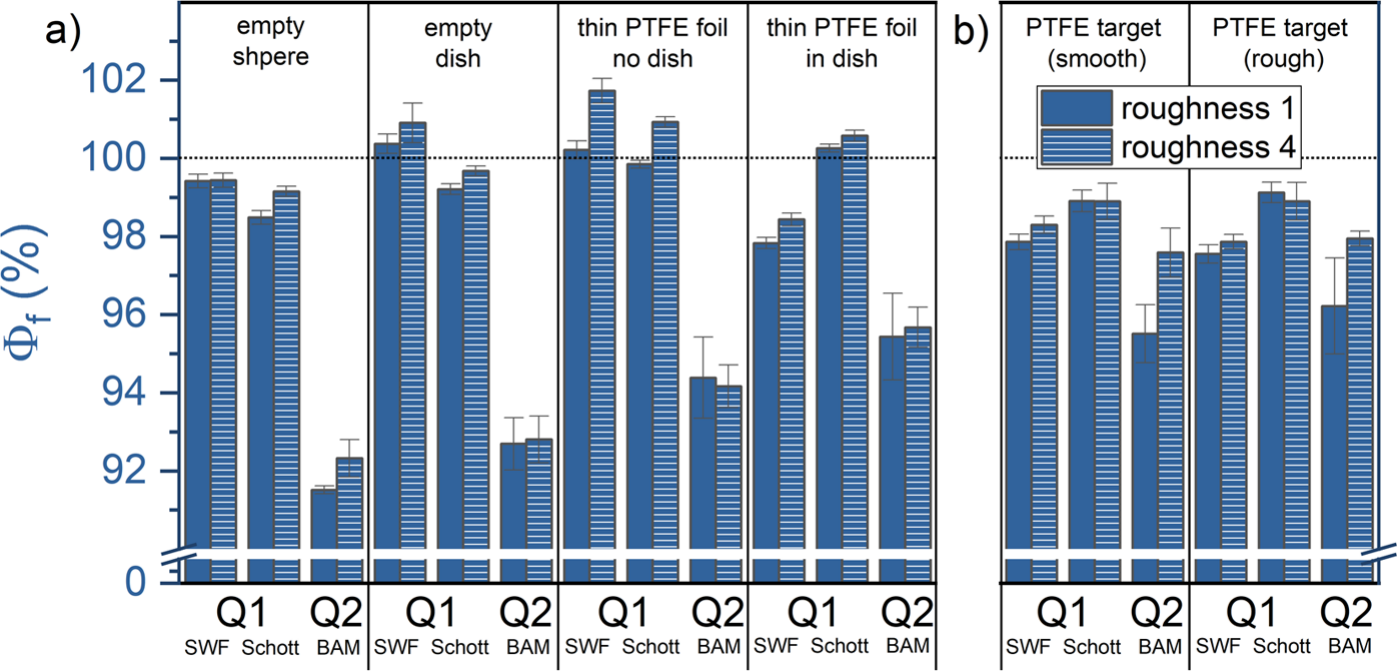
(a) Φ_f_ values of YAG:Ce OC samples without (solid blue) and with the highest degree of surface roughness (striped), determined with IS setups Q1 and Q2 using four different blanks: (i) empty IS, (ii) empty Petri dish, (iii) thin PTFE foil without the Petri dish, and (iv) thin PTFE foil in the Petri dish. (b) Φ_f_ measurements utilizing a 2 mm thick PTFE target with a smooth and a rough surface roughness as a blank. λ_exc_ = 450 nm.

To assess the influence of the sample’s surface properties on the Φ_f_ determination with Q2, four YAG:Ce OC samples with different surface roughness were produced. The Φ_f_ of these diffusely reflecting samples were then measured using four different blanks (Table S7). To study the effect of the specular reflecting surface of the quartz lid of the Petri dish and the diffusely scattering surfaces of the thin PTFE foil and the Spectralon surface of the IS in combination with different degrees of roughness of the YAG:Ce OCs, we selected the previously used thin PTFE foil with and without the Petri dish (without a lid), the empty IS, and the empty Petri dish without any additional blank material. The Φ_f_ values derived for the YAG:Ce OCs with a small and a high surface roughness (sample 1 with the smoothest and sample 4 with the highest surface roughness) are displayed in Figure 6a. The results obtained for samples 2 and 3 with intermediate surface roughness are shown in Figure S6 and summarized in Table S7. Interestingly, Figure 6a reveals that the surface roughness of the sample did not significantly affect the Φ_f_ values obtained with Q1 and Q2 regardless of the blank used. However, the Φ_f_ values clearly differed for both IS setups. As observed in the previous section, the blank considerably influences the Φ_f_ values obtained with both setups: (i) Using the PTFE foil with and without the Petri dish led to Φ_f_ values exceeding 100% for Q1, while with Q2, maximum Φ_f_ values of (95.7 ± 0.5)% were obtained. (ii) Similar results are found for the empty Petri dish with Φ_f_ exceeding 100% or reaching values close to 100% for Q1, while measurements with Q2 exhibited Φ_f_ values of (92.8 ± 0.6)%. (iii) While Φ_f_ measurements with the empty IS as a blank did not yield Φ_f_ values >100%, the deviation between the Φ_f_ measurements with Q1 and Q2 was found to be 7.2%. As previously suggested, these effects can be attributed to (i) differences in the light distribution of the scattering sample/blank as result of the differences in illumination and detection geometry for Q1 and Q2 and/or (ii) changes in the reflectivity of the IS surface at the sample position, potentially due to contaminations and/or aging effects. Apparently, none of the used blanks sufficiently match the samples’ scattering properties (specular or diffuse). Overall, the Φ_f_ values measured with Q1 seem to be overestimated, as indicated by Φ_f_ values exceeding 100%. For Φ_f_ measurements with Q2, all Φ_f_ values were <100% with deviations of 3.9% and 3.4% between the four different blanks (Figure 6a) for samples 1 and 4, respectively.

Then, we assessed the performance of a custom-made 2 mm thick PTFE target blank with a high (>95%) and almost wavelength-independent reflectivity in the visible. Due to its larger thickness, the reflectivity of the PTFE target is about 20% higher than that of the 250 μm thin PTFE foil (cf. Figure S7). To mimic the sample surface, one side of the PTFE foil was polished, and the other was kept “rough” for effective diffuse scattering. Figure 6b displays the Φ_f_ values of samples 1 and 4 obtained with the smooth and rough side of the PTFE foil. Generally, Φ_f_ values measured by the three ILC partners are (i) below 100% and (ii) more closely match, within the statistical uncertainties.

The different surface roughnesses of samples 1 and 4 did not affect the Φ_f_ values measured with Q1, yet deviated by about 2% for Q2, with the smoother sample 1 yielding smaller Φ_f_ values. For sample 4, similar Φ_f_ values were obtained as measured with Q1 for the smooth and rough PTFE target. Overall, our results indicate slightly higher experimental uncertainties of about 2% for sample 1 (smooth surface with a specular reflex) for Q2. Comparing the overall performance of the three IS setups, measurements with Q1 yielded higher Φ_f_ values with smaller experimental uncertainties. However, Φ_f_ values exceeding 100% were measured depending on the chosen blank. Measurements with Q2 led to smaller Φ_f_ values with slightly higher uncertainties, which are more strongly affected by the blank.

These deviations cannot be attributed to instrument calibrations (cf. Figure 2) but are likely caused by the measurement geometry and the light distribution inside the IS. This is supported by converging Φ_f_ values measured with Q1 and Q2 with a nonabsorbing diffusely reflecting target (PTFE, 2 mm) blank.

During the ILC, we noticed that some deviations in Φ_f_ measurements may result not only from instrument calibration and sample handling but also from the instrument settings, i.e., the accuracy of the automatically selected measurement parameters by the instrument and their stability. This can differ between IS setups. An example is shown in Figures S8–S10, where the spectral shape of the excitation peak shifted between sample and blank measurements, pointing to lamp instabilities or a monochromator drift. For the measurement series with *N* = 4 × 2 × 4, the Φ_f_ values are solely affected by handling uncertainties. Also instrument aging must be considered as revealed by differences in Φ_f_ values of about 2% for the same sample/blank pair, which were collected five months apart.

## Conclusion and Outlook

Aiming for the identification and quantification of uncertainty sources of Φ_f_ measurements of scattering liquid and solid luminescent samples, we performed the first interlaboratory comparison (ILC) of the absolute Φ_f_ measurements. This ILC involved three laboratories from academia and industry, using two commercial stand-alone integrating sphere (IS) setups with different illumination and detection geometries (Quantaurus first (Q1) and second (Q2) generation from Hamamatsu). As representative samples, we selected transparent and scattering dye solutions as well as solid phosphors, namely YAG:Ce optoceramics (YAG:Ce OC) broadly exploited as converter materials for blue LEDs. Following carefully developed measurement protocols for the ILC, we systematically explored (i) the influence of the illumination and detection geometry, (ii) the optical properties of the blank, necessary to determine the number of absorbed photons, and (iii) the influence of the sample-specific surface roughness, representatively varied for YAG:Ce OC samples. As a prerequisite for the ILC, first, the reliability of the spectral correction curves implemented by the instrument manufacturer was assessed using the BAM-certified spectral fluorescence standards F003–F005. The good agreement of the measured corrected emission spectra and certified spectra with dye spectra obtained in a previous ILC on the spectral correction of emission spectra confirmed the reliability of the setup calibrations.

One of the key findings of this ILC is that although the differences in the illumination and detection geometry of the two IS setups appear to be small, they are only negligible for liquid transparent luminescent samples. These differences appear to be also insignificant for scattering luminescent solutions, yet a prerequisite is a blank with scattering properties closely matching those of the sample. This was realized in this ILC by matching the concentrations of the silica particles in the dye solutions and the blank. For a more general statement and general recommendations for the absolute determination of the Φ_f_ of scattering luminescent dispersions, comprehensive experiments with luminescent particle dispersions covering a large range of scattering cross sections and scatterer concentrations are necessary, which was beyond the scope of this ILC.

For absolute Φ_f_ measurements of solid luminescent and scattering materials such as broadly applied OCs, special care is needed to circumvent measurement uncertainties originating from impurities or changes in the light distribution caused by different scattering and reflection properties of the blank and the sample. Reliable absolute Φ_f_ measurements of such samples require careful consideration of the illumination and detection geometry of the IS setup and the selection of a suitable blank. Criteria for a blank choice are (i) a good match with the samples’ scattering properties, specular or diffuse, and (ii) the ease of handling and reproducible positioning within the integrating sphere. Use of the sample holder as a blank is not recommended, due to potential aging of its material and the ease of introducing absorbing and/or emissive contaminations from previously measured samples. Also, BaSO_4_ powders and thin PTFE foils cannot be recommended as blanks for such OCs or other solid samples with similar scattering characteristics. Some of the BaSO_4_ powders utilized in this ILC led to an overestimation of the resulting Φ_f_ by about 20%, indicating batch-to-batch variations of commercial BaSO_4_ powders. Moreover, BaSO_4_ powders can introduce larger uncertainties related to handling, as their surface roughness cannot be well-controlled and reproduced. Also, the use of thin and flexible PTFE foil blanks can lead to considerable deviations between the Φ_f_ data obtained with different IS setups. Ultimately, we recommend nonabsorbing blank materials with a high reflectivity (>95%) like the 2 mm thick PTFE target to be placed on the sample holder, as this provided physically meaningful and comparable Φ_f_ values for both IS setups used in this ILC. We ascribe this finding to the near-Lambertian light scattering behavior of this material, yielding a homogeneous light distribution within the IS.

Overall, standardized measurement protocols in combination with a validated blank are mandatory to ensure reliable Φ_f_ measurements. Only this will enable a direct comparison of different IS setups and Φ_f_ data from different laboratories. In the future, we plan a similar ILC with a larger number of partners with different types of IS setups to broadly establish measurement uncertainties and identify instrument-specific sources of uncertainty. Also, scattering Φ_f_ standards were developed by BAM.

## Supporting Information Available

Additional experimental details, calculation of uncertainty contributions and uncertainty budget, method, validation, and overview of CRM spectral properties (PDF)
