# Supplementary material for: Interlaboratory Comparison on Absolute Photoluminescence Quantum Yield Measurements of Solid Light Converting Phosphors with Three Commercial Integrating Sphere Setups
Source: Anal Chem. 2024 Apr 17;96(17):6730–7. doi: 10.1021/acs.analchem.4c00372 (PMC11063975; doi:10.1021/acs.analchem.4c00372)
Supplement: Supplementary file 1 — ac4c00372_si_001.pdf [file ac4c00372_si_001.pdf]

Supporting Information

Interlaboratory comparison on absolute photoluminescence quantum yield measurements of solid light converting phosphors with three commercial integrating sphere setups

Saskia Fiedler[‡,a,b], Florian Frenzel[‡,a], Christian Würth [a], Isabella Tavernaro [a], Michelle Grüne [c], Stefan Schweizer [c,d], Axel Engel [e], and Ute Resch-Genger [a]*.

[a] Division *Biophotonics*, Federal Institute for Materials Research and Testing (BAM), Richard-Willstaetter-Strasse 11, D-12489 Berlin, Germany; email: ute.resch@bam.de

[b] Present address: Photonic Materials, NWO-Institute AMOLF, Science Park 104, 1098 XG Amsterdam, The Netherlands

[c] Faculty of Electrical Engineering, South Westphalia University of Applied Sciences, Lübecker Ring 2, 59494, Soest, Germany

[d] Fraunhofer Application Center for Inorganic Phosphors, Branch Lab of Fraunhofer Institute for Microstructure of Materials and Systems IMWS, Lübecker Ring 2, 59494, Soest, Germany

[e] Schott AG *Technical Services*, Hattenbergstrasse 10, D-55122 Mainz, Germany

Table of content

1. Sample preparation ……………………………………………………………………………………………………...S2
2. Φ_f – Measurements …………………………………………………………………………………………………... S2
3. Synthesis of SiO_2 particles ……………………………………………………………………………………………...S5
4. Additional optical information on OC samples ………………………………………………………………………S6
5. Literature …… S11

1. Sample preparation

The transparent ethanolic solutions of the spectral fluorescence standards F003, F004, and F005 were prepared with an absorbance (OD) of 0.1 at the dye absorption maxima of 377 nm, 423 nm, and 553 nm, respectively. For the studies with known amounts of 300 nm sized SiO_2 particles added as scatterers, concentrations of OD 0.1 were used for the dyes F016 (λ_{exc} = 400-420 nm), F017 (λ_{exc} = 530-550 nm), and F019 (λ_{exc} = 360-380 nm). The dye concentration of Φ_f standard F015 was adjusted to an OD of 0.06 at 500 nm.[1] All solutions were then diluted and re-measured to eliminate potential concentration-related reabsorption effects. For the preparation of scattering dye solutions, always the same amount of 300 nm sized SiO_2 particles (1.04 ml) from a stock solution (1.2 g/l SiO_2 particles in ethanol) was added to the ethanolic dye and blank solutions (each 1.47 ml). Prior to each measurement, a vortex mixer was used to ensure that the SiO_2 particles did not sediment before or during the experiment. Polycrystalline, transparent YAG:Ce optoceramics (OCs) were fabricated by a solid-state reaction method using high-purity Al_2O_3 and Y_2O_3 powders. The mixed powder compacts were sintered at temperatures above 1600 °C in vacuum for several hours.[2] To also assess the influence of the sample-specific parameter surface roughness, the YAG:Ce OC sample was roughened using water based SiC slurry with SiC grain sizes of 15 μm, 25 μm, 45 μm, and 60 μm, respectively.

2. Φ_f - Measurements

All interlaboratory comparison (ILC) measurements were performed using same measurement protocols on the exact same samples. The data analysis of the separately collected blank/reference and sample spectrum was automatically done by each instrument; general details on the analysis can be found elsewhere[3-7]. *Liquid samples*: All transparent dye solutions and all dye-particle dispersions were placed in a long-neck cuvette and measured twice in the same position, followed by a turn of 90 degrees for the next measurements, stepwise covering all four sides of the cuvette. This procedure, together with a new blank measurement of pure ethanol (with and without scatterers depending on the sample), was repeated three times (N = 4x2x3). *Solid samples*: For the measurements of the solid samples, the OC was placed on the sample holder in a quartz petri dish (without a lid). Data was collected twice at the same sample position before rotating the petri dish by about 90 degrees to re-measure. This was repeated three times to allow a full rotation of 360 degrees (N = 4x2x4). Subsequently, this procedure was repeated for a new blank measurement. To exclude uncertainties by transport-related changes of the samples, all ILC samples were returned to the respective provider, i.e., BAM and Schott, after the ILC and controlled.

Table S1. Differences of integrating sphere (IS) setups Quantaurus 1 (Q1): C9920-02G (FH SWF and Schott) and Quantaurus 2 (Q2): C11347-11 (BAM).

Parameter/quantity	Quantaurus 1 (C9920-02G)	Quantaurus 2 (C11347-11)
Participant	FH SWF / Schott	BAM
Light coupling	Fiber	Free space
Sphere diameter	8.38 cm	
Sphere coating	Spectralon	
Detector unit	CCD (300 – 950 nm)	
Light source bandwidth	about. 2 - 5 nm	10 nm or less (FWHM)
Excitation-detection angle	90-degree angle	28-degree angle
Long-neck cuvette position used for solutions	Center mounted, 90-degree to exc. and collection	Center mounted, 90-degree to exc., 28-degree to collection
Laboratory dish position for solids	Bottom, 8 degrees tilted to exc., 45 degrees to collection	Bottom, perpendicular to exc., 53-degrees to collection
Reab. Cor.	No	Yes

Table S2. Averaged (N = 4x2x3) absolutely measured F_f of transparent solution of the certified quantum yield standards F015 (λ_{ex} = 500 nm), F016 (λ_{exc} = 400-420 nm), F017 (λ_{exc} = 530-550 nm), and F019 (λ_{exc} = 360-380 nm) using Q1 and Q2. For the determination of the F_f values of F015, re-absorption corrections were performed.*[1]

Dye	BAM (%)	FH SWF (%)	Schott (%)	Certificate* (%)
F015	91.8±1.6	94.5±2.7	93.4±0.8	96±5.0
F016	56.1±0.2	58.4±2.0	57.2±1.7	59±4.0
F017	63.0±0.4	67.4±3.5	63.9±2.5	61±3.0
F019	53.2±0.3	53.8±1.9	55.0±3.3	53±4.0

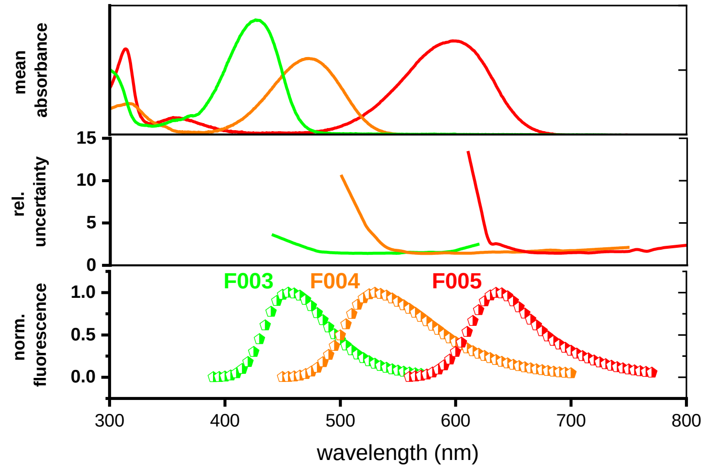

Figure S1 Absorption spectra (top), relative uncertainties (middle), and normalized spectrally corrected fluorescence spectra (bottom) of the BAM-certified spectral fluorescence standards, i.e., the KIT dyes F003-F005 provided by BAM for the control of the reliability of the emission correction curves of the IS setups Q1 and Q2 implemented by the instrument manufacturer.

Table S3 Averaged (N = 4x2x3) absorption values of absolute F_f measurements of transparent solutions (certified reference materials F015, F016, F017 and F019) in long-neck cuvette using the different IS setups Q1 (FH SWF and Schott) and Q2 (BAM). For the values of F015 a re-absorption correction was performed.

Dyes	Absorption in %					
	BAM		FH SWF		Schott	
	OD 0.06		OD 0.06		OD 0.06	
F015 (λ_{exc} = 500 nm)	34.8±0.1		39.7±0.8		44.8±2.4	
	OD 0.1	OD 0.05	OD 0.1	OD 0.05	OD 0.1	OD 0.05
F016 (λ_{exc}= 400-420 nm)	46.5±0.4	29.6±0.1	50.5±0.5	30.4±3.5	54.5±3.6	28.5±1.0
F017 (λ_{exc}= 530-550 nm)	46.4±0.1	29.6±0.1	53.6±1.5	32.8±1.5	53.3±1.7	21.4±0.9
F019 (λ_{exc}= 360-380 nm)	40.8±0.3	25.3±0.1	42.3±0.8	25.5±2.8	43.6±4.5	29.5±1.9

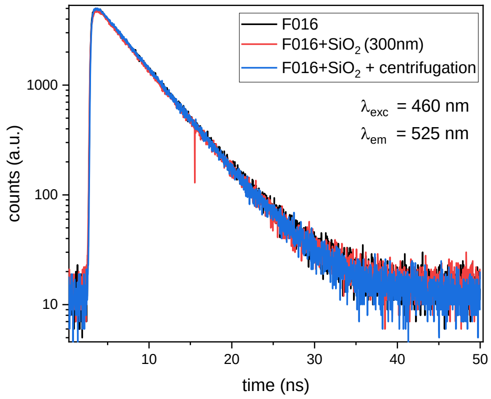

Figure S2 Fluorescence decay kinetics of the BAM F_f standard dye F016 measured without 300 nm SiO_2 particles and obtained in the presence of and with 300 nm SiO_2 particles. The excellent of the match of the fluorescence decay curves of the dye alone, in the presence of the scattering SiO_2 particles, and after purification via multiple centrifugation steps illustrates that the SiO_2 particles do not affect the fluorescence decay kinetics of dye F016 and thus also not on its Φ_f value. This confirms the absence of fluorescence quenching.

Table S4 Absolutely measured Φ_f of F015 in solution without and with SiO_2 particles in long-neck cuvette collected with IS setups Q1 and Q2. An additional re-absorption correction was performed due to a small Stokes shift and therefore spectral overlap of absorption and fluorescence signal.

**Sample**	**Blank**	**Institute**	**Exc. wavelength**	**Avg Φ_f (%)**	**Avg Abs (%)**	**Re-absorption-Corrected Φ_f (%)**
F015	EtOH	BAM	500 nm	90.3±2.0	34.8±0.1	**91.8±1.6**
F015+SiO_2	EtOH+SiO_2	BAM	500 nm	92.5±0.2	27.3±0.3	**93.4±0.4**
F015	EtOH	FH SWF	500 nm	92.8±2.7	39.7±0.8	**94.4±2.7**
F015+SiO_2	EtOH+SiO_2	FH SWF	500 nm	94.6±2.2	31.2±0.5	**95.6±0.2**
F015	EtOH	Schott	500 nm	92.2±1.5	44.8±2.4	**93.4±0.8**
F015+SiO_2	EtOH+SiO_2	Schott	500 nm	91.3±1.0	24.3±0.6	**91.9±0.6**

Table S5 Absolutely measured Φ_f of F015 in solution without SiO_2 particles using long-neck cuvettes and setups Q1 and Q2. The dye concentration was set to values yielding absorbances (OD) between 0.02 – 0.03 at the chosen excitation wavelength.

**Sample**	**OD (meas.)**	**Institute**	**Exc. Wave-length**	**Avg Φ_f (%)**	**Corrected* Φ_f (%)**	**Avg Abs (%)**
F015	0.02	BAM	500 nm	92.2±0.7	92.6±0.7	5.5±2.2
F015	0.03	FH SWF	500 nm	94.8±4.4	95.5±4.4	17.8±0.7
F015	0.02	Schott	500 nm	92.3±1.5	92.9±1.4	18.4±0.8

3. Synthesis of SiO_2 particles

In this ILC, in-house synthesized SiO_2 particles with a primary particle size of 300 nm were utilized. The amorphous, non-porous SiO_2 sub-microparticles were prepared according to the well-known Stöber sol-gel approach in a mixture of EtOH and doubly distilled water (MilliQ-water, 0.055 µS m^{-1}) using ammonia (NH_4OH) as a catalyst and tetraethyl orthosilicate (TEOS) as the silicium precursor.[8,9]

To obtain silica particles with a primary particle size of 300 nm, 33 ml of absolute EtOH (99.9%, *Labsolute*) were mixed with 6.65 ml of MilliQ-water and stirred for 5 min at 45 °C. 2.3 ml of NH_4OH (25%, *Merck*) was added to the mixture, before 3.35 ml ($1.51 \cdot 10^{-5}$ mol) of TEOS (≥99.0%, *Sigma Aldrich*) was added. The reaction mixture was stirred at 45 °C overnight. The particles were purified by centrifugation (16,000 rcf, 15 min), washed thrice with a mixture of EtOH:MilliQ-water (3:1; 1:1; 1:3), and stored in EtOH with a concentration of 3 mg/ml.

The physicochemical particle properties size, shape, state of agglomeration, and surface charge were characterized via transmission electron microscopy (TEM), dynamic light scattering (DLS), nanoparticle tracking analysis (NTA), and zeta potential measurements. NTA was carried out with a NanoSight LM 10 system of *Malvern Panalytical* equipped with a 405 nm laser at a temperature of 25 °C. Here for each sample 5 videos with 60 s and 25 fps were recorded to calculate the number-based hydrodynamic diameter of the particles and their concentration. DLS and zeta potential measurements were performed using a *Malvern Panalytical* Zetasizer Nano ZS equipped with a 630 nm laser at 25 °C. All particles were dispersed in MilliQ-water for these measurements. The zeta potential was calculated from the nanoparticle electrophoretic mobility using the Einstein-Smoluchowski theory. The number-based hydrodynamic diameter was measured at back scattering angle 173° with a total measurement time of 10 min, followed by calculation using a refractive index of 1.4649 for SiO_2 particles. The morphology, average particle diameter, and the agglomeration state of the particles were determined using a Tecnai G2 20 S-Twin from *FEI*. TEM measurements were carried out in part at the electron microcopy center at BAM. The particles were ultrasonicated for the measurements, diluted, and drop casted onto a TEM grid. The particle size distribution was representatively determined for a randomly chosen sample of 0-150 particles using the X-ImageJ software (Version: 1.52 e, winPenPack X-ImageJ Launcher from the National Institute of Health (http://rsb.info.nih.gov/ij/).

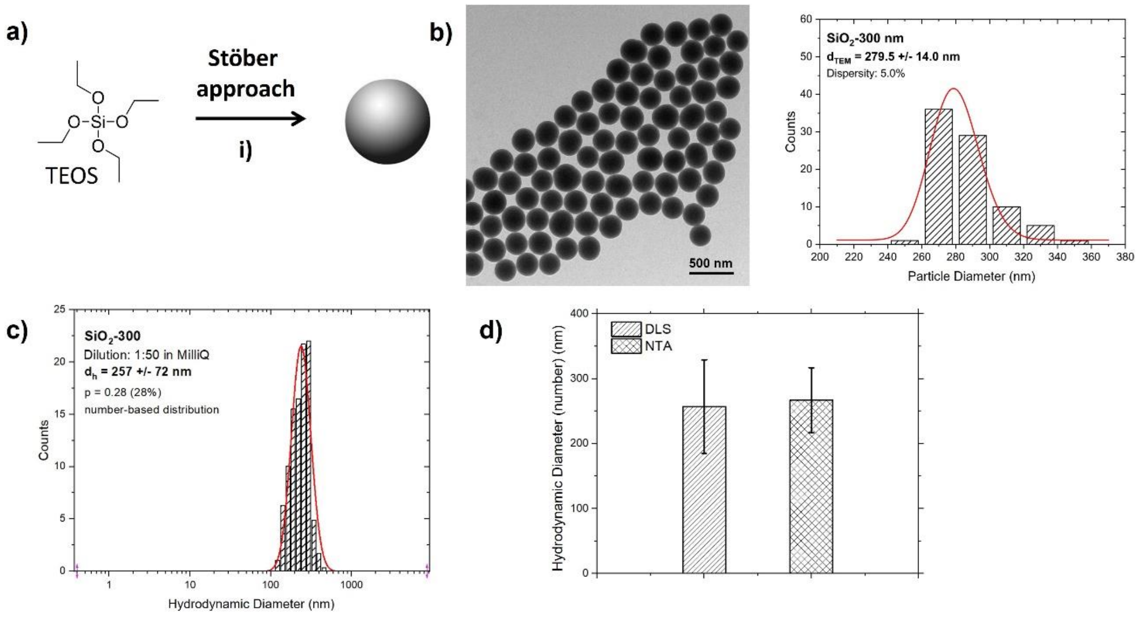

Figure S3 Reaction scheme of the synthesis of 300 nm sized SiO_2 particles as scatters using Stöber approach, i) NH_4OH, EtOH/MilliQ-water, 45 °C, overnight (a). The TEM micrograph indicates single spherical particles with a narrow size distribution of 5% and a primary particle size of 279.5 nm (b). The number-based hydrodynamic diameter obtained by DLS measurements shows a higher size distribution with an average size of 257 ± 72 nm (c), that was confirmed by NTA measurements (d) with a number based hydrodynamic diameter of 257 ± 50 nm and a particle concentration of $4 \cdot 10^{11}$ particles/ml. Zeta potential measurements resulted in very stable, negatively charged particle suspensions with a zeta potential of -46.3 ±0.6 mV.

4. Additional optical information on OC samples

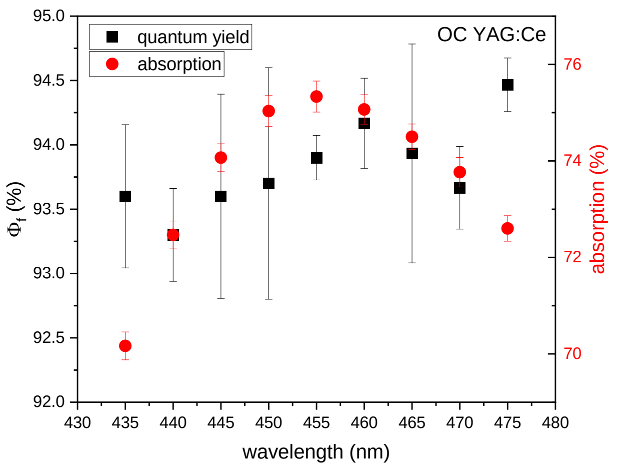

Figure S4 YAG:Ce OC: Excitation wavelength-dependent of Φ_f (black) and corresponding absorption (red) values. A thin PTFE foil was used as a blank.

Table S6 Absolutely measured Φ_f of YAG:Ce OC sample, with $BaSO_4$ powder and a thin PTFE foil as blank collected at the three IS setups at BAM, FH SWF and Schott.

**Sample**	**Blank** (quartz dish w/o lid)	**Institute**	**Exc. wavelength**	**Avg Φ_f (%)**	**Avg Abs (%)**
YAG:Ce OC	thin PTFE foil	BAM	450 nm	93.6±0.9	75.0±0.7
YAG:Ce OC	thin PTFE foil	FH SWF	450 nm	99.5±0.3<br>99.7±0.4*	86.7±0.2<br>86.7±0.2
YAG:Ce OC	thin PTFE foil	Schott	450 nm	99.5±0.4<br>99.6±0.4*	86.9±0.1<br>86.9±0.1*
YAG:Ce OC	$BaSO_4$ powder	BAM	455 nm	98.5±1.4	65.0±0.9
YAG:Ce OC	$BaSO_4$ powder	FH SWF	450 nm	98.9±0.4<br>99.2±0.7*	82.2±0.4<br>82.1±0.5*
YAG:Ce OC	$BaSO_4$ powder	Schott	450 nm	99.4±0.7<br>99.6±0.7*	82.5±0.6<br>82.4±0.6*

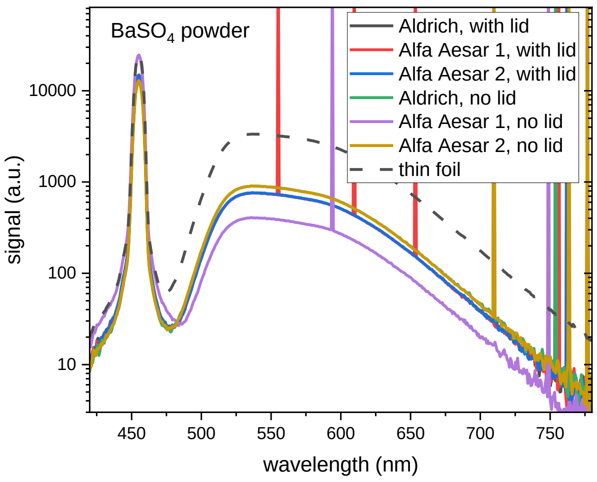

Figure S5 Emission spectra of the YAG:Ce OC sample using different $BaSO_4$ powders from Sigma Aldrich and Alfa Aesar (1 (B2) and 2 (B3)), with and without the lid of the petri dish. Sharp spikes are caused by cosmic rays.

Table S7 Averaged Φ_f and absorption values of YAG:Ce OC sample with variation of surface roughness (low to high roughness from sample 1 to sample 4) and variation of thin PTFE foil blank positions (thin PTFE foil without the petri dish, empty IS, thin PTFE foil in petri dish without lid, and empty petri dish without an additional blank material) for all four samples collected with Q1 (FH SWF and Schott) and Q2 (BAM).

**Blank (excited @450 nm)**	**Institute**	**Avg Φ_f (%): Sample 1**	**Avg Abs (%): Sample 1**	**Avg Φ_f (%): Sample 2**	**Avg Abs (%): Sample 2**	**Avg Φ_f (%): Sample 3**	**Avg Abs (%): Sample 3**	**Avg Φ_f (%): Sample 4**	**Avg Abs (%): Sample 4**
thin PTFE foil as blank, no cuvette	BAM	94.4±1.0	73.1±1.5	94.8±0.8	74.0±1.0	93.5±0.6	73.1±0.8	94.2±0.5	74.4±1.4
thin PTFE foil as blank, no cuvette	FH SWF	100.2±0.2	86.5±0.1	101.5±0.2	85.8±0.2	102.0±0.5	85.7±0.2	101.7±0.3	85.2±0.2
thin PTFE foil as blank, no cuvette	Schott	99.9±0.1	86.9±0.1	100.5±0.1	86.5±0.0	100.5±0.2	86.6±0.1	100.9±0.1	85.8±0.1
Empty sphere as blank	BAM	91.5±0.1	74.1±1.1	91.8±0.7	74.5±0.5	92.3±0.4	74.4±0.4	92.3±0.5	74.2±1.1
Empty sphere as blank	FH SWF	99.4±0.2	86.6±0.1	99.8±0.3	85.8±0.1	99.6±0.3	86.0±0.2	99.4±0.2	85.5±0.1
Empty sphere as blank	Schott	98.5±0.2	86.9±0.2	99.0±0.0	86.5±0.1	98.9±0.1	86.6±0.1	99.2±0.1	85.8±0.1
thin PTFE foil in cuvette, no cover	BAM	95.4±1.1	73.3±1.5	94.9±0.5	73.3±1.6	95.8±1.0	72.6±0.6	95.7±0.5	72.9±1.4
thin PTFE foil in cuvette, no cover	FH SWF	97.8±0.1	86.8±0.1	98.4±0.2	86.4±0.1	98.3±0.3	86.6±0.1	98.4±0.2	85.6±0.2
thin PTFE foil in cuvette, no cover	Schott	100.3±0.1	87.1±0.1	100.6±0.1	86.7±0.1	100.6±0.1	86.8±0.04	100.6±0.1	86.0±0.1
Empty cuvette as blank	BAM	92.7±0.7	74.7±0.8	92.6±1.1	74.4±0.9	92.9±1.0	74.1±1.6	92.8±0.6	74.2±1.2
Empty cuvette as blank	FH SWF	100.4±0.2	86.7±0.2	100.4±0.4	86.2±0.2	100.6±0.4	86.4±0.2	100.9±0.5	85.8±0.3
Empty cuvette as blank	Schott	99.2±0.1	86.9±0.0	99.7±0.1	86.5±0.0	99.5±0.2	86.6±0.1	99.7±0.1	85.7±0.1

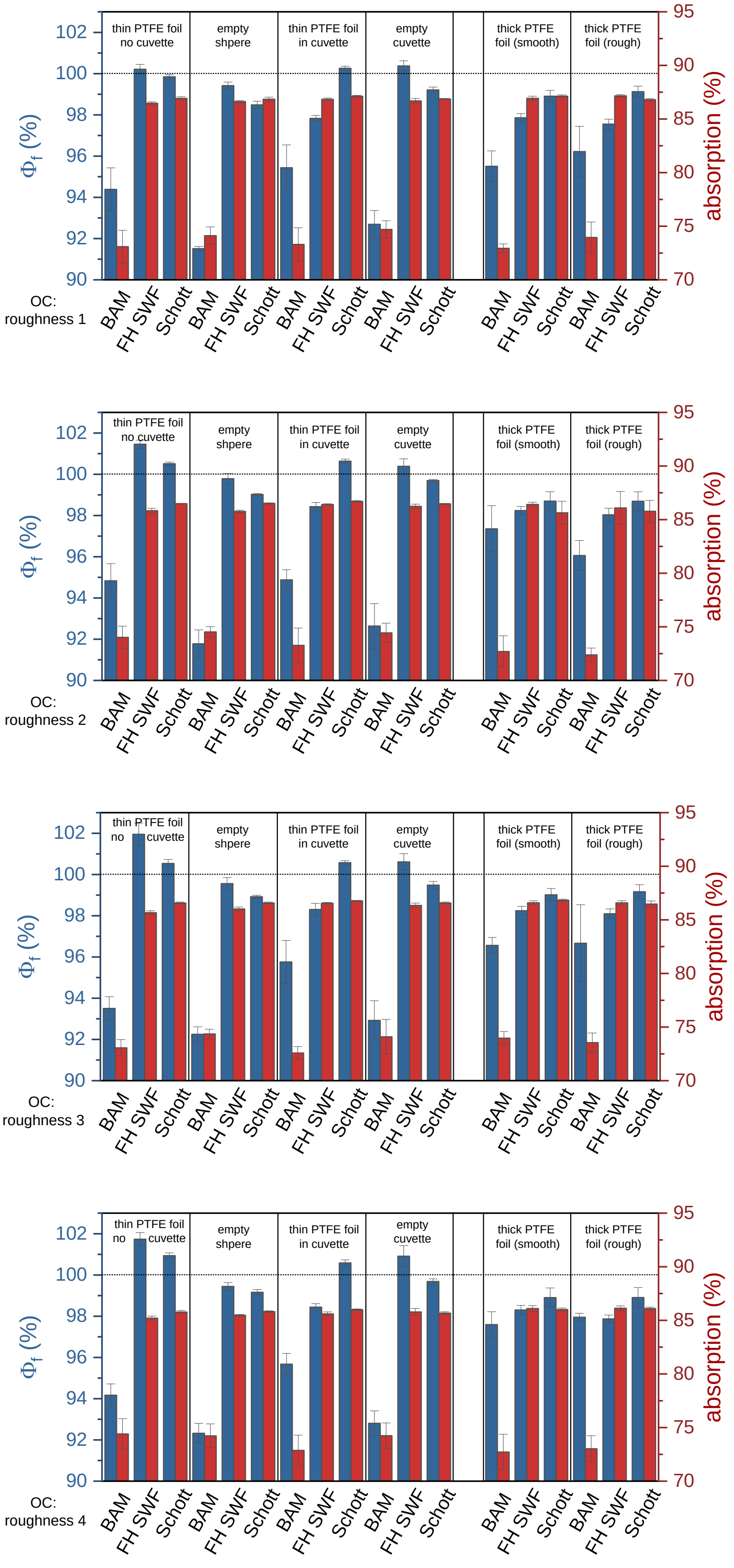

Figure S6 Comparison of absorption and Φ_f of YAG:Ce OC samples with increasing degree of surface roughness, excited at 450 nm. For all samples, four different blanks (thin PTFE foil without a petri dish, empty IS, thin PTFE foil in a petri dish, and empty petri dish without an additional blank material) and an additional 2 mm-thick PTFE foil with a smooth and rough surface side were used.

Table S8 Influence of the surface roughness of YAG:Ce OC and variation of PTFE foil blank surface side (rough and smooth).

Sample	Blank	Institute	Exc. wavelength	Avg Φ_f (%)	Avg Abs (%)
Sample 1	PTFE (rough side)	BAM	450 nm	96.2±1.2	74.0±1.4
Sample 1	PTFE (rough side)	FH SWF	450 nm	97.6±0.2	87.2±0.1
Sample 1	PTFE (rough side)	Schott	450 nm	99.1±0.3	86.8±0.1
Sample 2	PTFE (rough side)	BAM	450 nm	96.1±0.7	72.4±0.6
Sample 2	PTFE (rough side)	FH SWF	450 nm	98.0±0.3	86.1±1.5
Sample 2	PTFE (rough side)	Schott	450 nm	98.7±0.5	85.8±1.0
Sample 3	PTFE (rough side)	BAM	450 nm	96.7±1.9	73.6±0.9
Sample 3	PTFE (rough side)	FH SWF	450 nm	98.1±0.2	86.6±0.2
Sample 3	PTFE (rough side)	Schott	450 nm	99.2±0.3	86.5±0.3
Sample 4	PTFE (rough side)	BAM	450 nm	98.0±0.2	73.0±1.2
Sample 4	PTFE (rough side)	FH SWF	450 nm	97.9±0.2	86.1±0.2
Sample 4	PTFE (rough side)	Schott	450 nm	98.9±0.5	86.1±0.1

Sample	Blank	Institute	Exc. wavelength	Avg Φ_f (%)	Avg Abs (%)
Sample 1	PTFE (smooth side)	BAM	450 nm	95.5±0.7	72.9±0.4
Sample 1	PTFE (smooth side)	FH SWF	450 nm	97.9±0.2	86.9±0.2
Sample 1	PTFE (smooth side)	Schott	450 nm	98.9±0.3	87.1±0.1
Sample 2	PTFE (smooth side)	BAM	450 nm	97.4±1.1	72.7±1.5
Sample 2	PTFE (smooth side)	FH SWF	450 nm	98.2±0.2	86.4±0.2
Sample 2	PTFE (smooth side)	Schott	450 nm	98.7±0.5	85.6±1.1
Sample 3	PTFE (smooth side)	BAM	450 nm	96.6±0.4	74.0±0.6
Sample 3	PTFE (smooth side)	FH SWF	450 nm	98.2±0.2	86.6±0.2
Sample 3	PTFE (smooth side)	Schott	450 nm	99.0±0.3	86.8±0.1
Sample 4	PTFE (smooth side)	BAM	450 nm	97.6±0.6	72.7±1.7
Sample 4	PTFE (smooth side)	FH SWF	450 nm	98.3±0.2	86.1±0.3
Sample 4	PTFE (smooth side)	Schott	450 nm	98.9±0.5	86.0±0.1

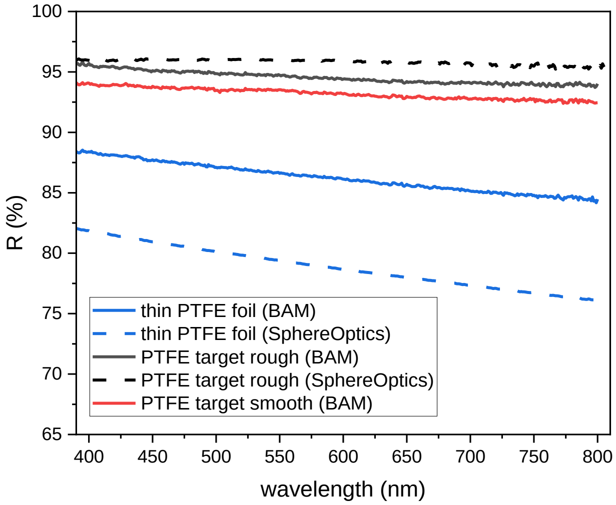

Figure S7 Wavelength-dependent reflection of the thick PTFE foil (d = 15 mm and t = 2 mm) – rough and smooth side – and of the thin PTFE foil.

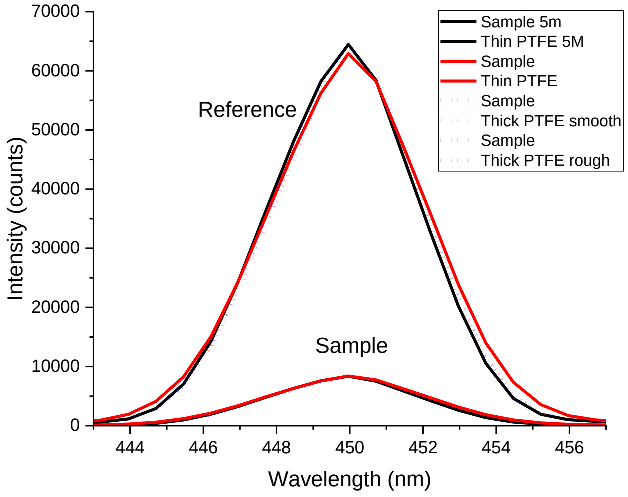

Figure S8 Reference/blank and sample measurement in the spectral range of excitation of OC using the rough and smooth side of the thick PTFE target and the thin PTFE foil as a blank. The measurements using the thin PTFE foil were repeated after five months (labeled '5M') resulting in a 2% deviation ($\Phi_{f,t=0}$ = 99.7 ± 0.4% and $\Phi_{f,t=5m}$ = 97.8 ± 0.1%). Using the smooth and rough side of the thick PTFE target resulted in Φ_f = 97.6 ± 0.2% and Φ_f =97.9 ± 0.2%, indicating that the absorption can be considered identical.

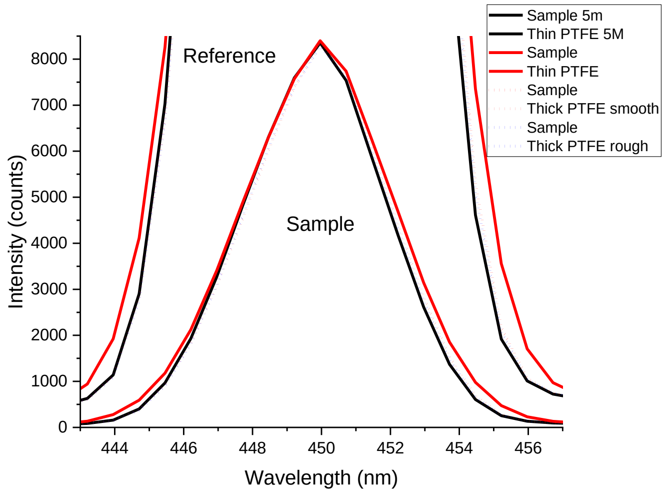

Figure S9 (Zoom of Figure S8) Reference/blank and sample measurement in the spectral range of excitation of OC using the rough and smooth side of the thick PTFE target and the thin PTFE foil as a blank. The measurements using the thin PTFE foil were repeated after 5 months (labeled '5M') showing the same 2% deviation ($\Phi_{f\ t=0}$ = 99.7 ± 0.4% and $\Phi_{f,t=\ 5m}$ = 97.8 ± 0.1%). Using the smooth and rough side of the thick PTFE resulted again in an unmeasurable difference in Φ_f (Φ_f = 97.6 ± 0.2% and Φ_f =97.9 ± 0.2%).

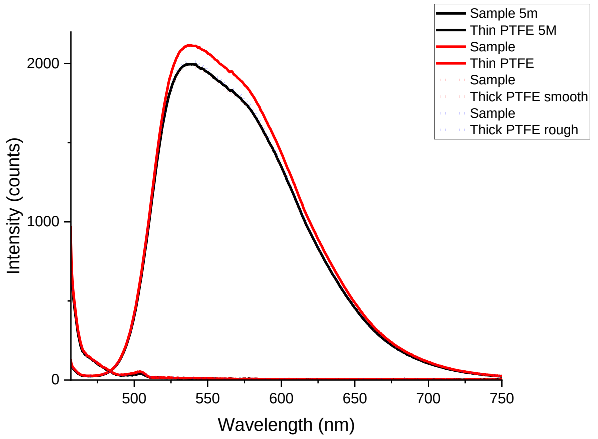

Figure S10 Reference/blank and sample measurement in the spectral range of emission of OC using the rough and smooth side of the thick PTFE target and the thin PTFE foil as a blank. The measurements using the thin PTFE foil were repeated after 5 months (labeled '5M') resulted in a 2% deviation ($\Phi_{f,t=0}$ = 99.7 ± 0.4% and $\Phi_{f,t=\ 5m}$ = 97.8 ±0.1 %). Using the smooth and rough side of the thick PTFE target resulted in Φ_f = 97.6 ± 0.2% and Φ_f =97.9 ± 0.2%.

Literature

(1) Pauli, J.; Güttler, A.; Schneider, T.; Würth, C.; Resch-Genger, U. *Analytical Chemistry* **2023**, *95*, 5671-5677.
(2) Ikesue, A.; Furusato, I.; Kamata, K. *Journal of the American Ceramic Society* **1995**, *78*, 225-228.
(3) Würth, C.; Resch-Genger, U. *Applied Spectroscopy* **2015**, *69*, 749-759.
(4) Würth, C.; Pauli, J.; Lochmann, C.; Spieles, M.; Resch-Genger, U. *Analytical Chemistry* **2012**, *84*, 1345-1352.
(5) Würth, C.; Lochmann, C.; Spieles, M.; Pauli, J.; Hoffmann, K.; Schüttrigkeit, T.; Franzl, T.; Resch-Genger, U. *Applied Spectroscopy* **2010**, *64*, 733-741.
(6) Würth, C.; Grabolle, M.; Pauli, J.; Spieles, M.; Resch-Genger, U. *Nature Protocols* **2013**, *8*, 1535-1550.
(7) Würth, C.; González, M. G.; Niessner, R.; Panne, U.; Haisch, C.; Genger, U. R. *Talanta* **2012**, *90*, 30-37.
(8) Stöber, W.; Fink, A.; Bohn, E. *Journal of Colloid and Interface Science* **1968**, *26*, 62-69.
(9) Ghimire, P. P.; Jaroniec, M. *Journal of Colloid and Interface Science* **2021**, *584*, 838-865.
